# Management of Lobular Neoplasia Diagnosed by Core Biopsy

**DOI:** 10.1155/2023/8185446

**Published:** 2023-04-18

**Authors:** Chinmay Jani, Margaret Lotz, Sarah Keates, Yasha Gupta, Alexander Walker, Omar Al Omari, Arshi Parvez, Dipesh Patel, Maria Gnata, John Perry, Leila Khorashadi, Lisa Weissmann, Susan E. Pories

**Affiliations:** ^1^Department of Internal Medicine, Mount Auburn Hospital, 300 Mount Auburn St., Cambridge, MA, USA; ^2^Harvard Medical School, 25 Shattuck St, Boston 02115, MA, USA; ^3^Division of Hematology-Oncology, Mount Auburn Hospital, 300 Mount Auburn St., Cambridge, MA, USA; ^4^Hoffman Breast Center, Department of Surgery, Mount Auburn Hospital, 300 Mount Auburn St., Cambridge, MA, USA; ^5^Department of Radiology, Mount Auburn Hospital, 300 Mount Auburn St., Cambridge, MA, USA; ^6^Department of Pathology, Mount Auburn Hospital, 300 Mount Auburn St., Cambridge, MA, USA

## Abstract

Lobular neoplasia (LN) involves proliferative changes within the breast lobules. LN is divided into lobular carcinoma in situ (LCIS) and atypical lobular hyperplasia (ALH). LCIS can be further subdivided into three subtypes: classic LCIS, pleomorphic LCIS, and LCIS with necrosis (florid type). Because classic LCIS is now considered as a benign etiology, current guidelines recommend close follow-up with imaging versus surgical excision. The goal of our study was to determine if the diagnosis of classic LN on core needle biopsy (CNB) merits surgical excision. This is a retrospective, observational study conducted at Mount Auburn Hospital, Cambridge, MA, from May 17, 2017, through June 30, 2020. We reviewed the data of breast biopsies conducted at our hospital over this period and included patients who were diagnosed with classic LN (LCIS and/or ALH) and excluded patients having any other atypical lesions on CNB. All known cancer patients were excluded. Of the 2707 CNBs performed during the study period, we identified 68 women who were diagnosed with ALH or LCIS on CNB. CNB was performed for an abnormal mammogram in the majority of patients (60; 88%) while 7(10.3%) had an abnormal breast magnetic resonance imaging study (MRI), and 1 had an abnormal ultrasound (US). A total of 58 patients (85%) underwent excisional biopsy, of which 3 (5.2%) showed malignancy, including 2 cases of DCIS and 1 invasive carcinoma. In addition, there was 1 case (1.7%) with pleomorphic LCIS and 11 cases with ADH (15.5%). The management of LN found on core biopsy is evolving, with some advocating surgical excision and others recommending observation. Our data show a change in diagnosis with excisional biopsy in 13 (22.4%) of patients with 2 cases of DCIS, 1 invasive carcinoma, 1 pleomorphic LCIS, and 9 cases of ADH, diagnosed on excisional biopsy. While ALH and classic LCIS are considered benign, the choice of ongoing surveillance versus excisional biopsy should be made with shared decision making with the patient, with consideration of personal and family history, as well as patient preferences.

## 1. Introduction

**Figure 1:**
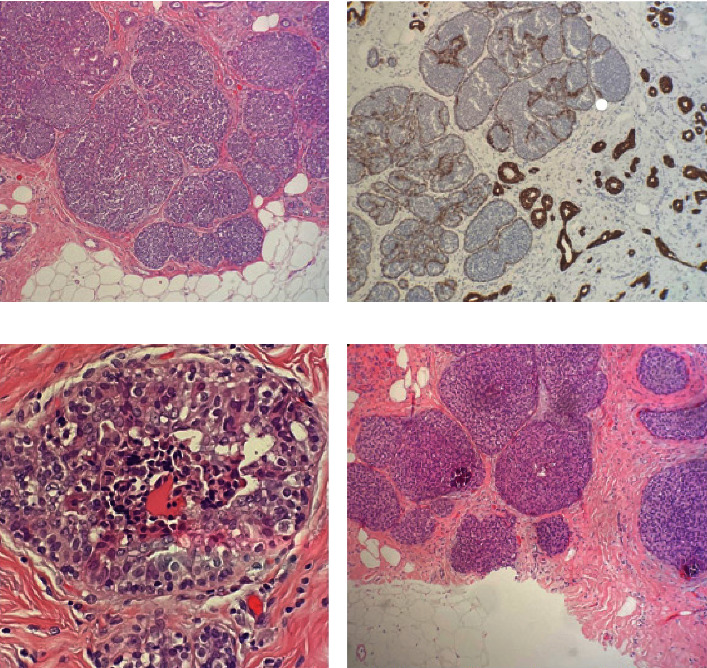
Classification of lobular carcinoma in situ. (a) Classic LCIS: H&E staining shows dyscohesive proliferation of epithelial cells in the duct lobular unit indicating lobular carcinoma in situ; more than 50% of the acini are filled and expanded by the neoplastic cells. (b) Classic LCIS: E-cadherin stain showing loss of membrane expression of the adhesion molecule E-cadherin, which is a signature feature of LCIS classic subtype. (c) Pleomorphic LCIS: H&E staining shows proliferation of dyscohesive cells with nuclear pleomorphism (enlarged nuclei, increased nuclear-to-cytoplasmic ratio, and prominent nucleoli). (d) Florid LCIS: H&E stain showing florid LCIS with marked distension of ductal lobular unit creating a confluent mass-like architecture. This demonstrates one of the classic features of florid LCIS of almost no intervening stroma between markedly distended acini of the involved units.

Lobular neoplasia involves proliferative changes within the breast lobules. This finding is classified as ALH or LCIS based on lobular involvement (>50% of the lobular region involved in LCIS), degree of lobular distension (more than benign lobules), and extent of lobule involvement (more than 50% acini involved in LCIS), and often the acinar lumen is occluded in LCIS. LCIS is subdivided into three subtypes: classic LCIS, pleomorphic LCIS, and florid LCIS (Figure 1). Pleomorphic LCIS and florid LCIS display solid proliferation of dyscohesive neoplastic cells within terminal duct lobular units (TDLUs) similar to classic LCIS but differ with regard to the degree of nuclear atypia and/or lobular acinar expansion [1]. Genomic profiling of different types of LCIS showed that pleomorphic LCIS and florid LCIS contain genetic alterations characteristic of lobular neoplasia; however, these variants are distinguished from classic LCIS reported in the literature by their highly recurrent ERBB2 alterations [2].

LCIS was first defined in 1941, and since then the status of LCIS has changed from a premalignant lesion to a risk marker, meaning that patients diagnosed with LCIS are at increased risk of developing invasive breast cancer [3, 4]. Management guidelines for LN are evolving and vary across institutions. As per the 2017 American Joint Commission of Cancer staging manual, classic LCIS is now considered as a benign lesion. Current NCCN guidelines state that a core biopsy showing classic LCIS can be managed without surgical excision, but that excision should be considered on a case-by-case basis. When surgical excision is not performed, close follow-up with mammogram and breast MRI screening every 6 to 12 months is recommended [5, 6]. Patients are also counseled regarding breast cancer risk reduction strategies such as chemoprevention [7].

Concerning features such as a high degree of atypia or necrosis seen on core needle biopsy (CNB) favor surgical biopsy for a full evaluation of the tissue [8]. In addition, the degree of concordance between the CNB and previous imaging studies can help guide further management; histopathological results that are inconsistent with the mammographic appearance of the lesion would favor surgical excision [8]. Pleomorphic LCIS or florid LCIS with marked nuclear pleomorphism is of greater concern due to the potential to progress to infiltrating pleomorphic lobular carcinoma and, as such, is treated like ductal carcinoma in situ and warrants surgical excision with clean margins and postoperative radiation [7–10]. Multiple foci LCIS (LCIS involving 4 terminal ductal units on CNB) is also associated with an increased risk of malignancy and warrants excisional biopsy [8].

The aim of this study was to examine the results of surgical excision after core biopsy showing the diagnosis of ALH or classic LN by breast core needle biopsy (CNB) to determine the frequency of change in diagnosis with resection of additional tissue.

## 2. Methods

This is a retrospective observational study that was conducted at Mount Auburn Hospital, Cambridge, MA, from May 17, 2017, until June 30, 2020. Institutional Review Board approval for chart review was obtained. We reviewed the data of 2707 breast CNBs conducted at our hospital and included all patients who were diagnosed with LN only on CNB (classic LCIS and/or ALH). Patients were excluded if the CNB showed invasive carcinoma, ductal carcinoma in situ (DCIS), pleomorphic or florid LCIS, a radial scar, ADH (atypical ductal hyperplasia), other atypia, or papillary lesions. Cases with radiologic-pathologic discordance were also excluded because these cases would routinely be excised. In addition, cases where the diagnosis of ALH or LCIS was made on breast reduction or patients having history of concurrent or previous breast carcinoma were excluded. Clinical information including the age, gender, race, and ethnicity was collected from our electronic medical records. Details of imaging were also collected. Results of the excisional biopsy were reviewed by the pathologist and recorded.

## 3. Results

We identified 68 female patients who were diagnosed with lobular neoplasia on CNB (Table 1). Our population's mean age was 55 years old, and the majority of patients were 50 years or older (47; 69%). The mean body mass index (BMI) was 25.7. The majority (78%) of patients were Caucasian, 5.9% were Asian, 4.4% were African American, and 11.8% did not have documentation of race. Most of the patients were non-Hispanic (86.8%). Patients with a personal history of breast cancer were excluded from the study as per our Methods section. However, a substantial number of the patients did have a family history of breast cancer: 26% had a first degree relative (mother or sister) with breast cancer and 60% had a second or third degree relative with breast cancer. The majority of these patients, 42 (72%), had no history of prior biopsy, 11 had 1 prior biopsy (19%), 4 (7%) had 2 prior biopsies, only 1 (2%) had more than 2 prior biopsies.

CNB was performed for an abnormal mammogram in the majority of patients (60; 88.2%), while 7 (10.3%) had an abnormal breast magnetic resonance imaging study (MRI) and 1 (1.5%) had an abnormal ultrasound. All patients had one site biopsied. Our radiologists' philosophy is to biopsy the most suspicious site and then plan for management of additional sites, if any, based on the results of the first biopsy.

A total of 58 patients (85.3%) underwent excisional biopsy, of which 3 (5.2%) showed malignancy, including 2 cases of DCIS and 1 invasive carcinoma (Table 2). In addition, 1 case (1.7%) with pleomorphic LCIS and 9 cases with ADH (19%) were identified on excisional biopsy. Ten patients with LN on CNB decided against surgical intervention (Table 3).

Out of the 3 patients found to have cancer on surgical excision, 2 of these had routine screening mammograms, while 1 was undergoing six-month follow-up evaluation. Of note, all 3 patients had a positive family history of breast cancer. All 3 patients were found to have calcifications raising concerns for CNB. On CNB, 2 patients showed ALH, while another patient showed LCIS. On excisional biopsy, both the patients with ALH were found to have DCIS, while the patient with LCIS showed multifocal invasive carcinoma (Table 2).

**Figure 2:**
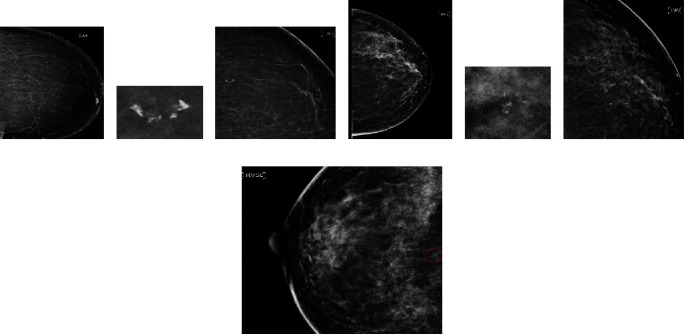
Radiographic findings. Representative radiographs from case of LCIS upgraded to invasive carcinoma at surgical excision. All calcifications in these cases were defined as “coarse and heterogenous,” which are by definition a BI-RADS 4B lesion and thus have a 10–50% risk of malignancy. There is no radiological finding that can reliably differentiate LCIS from more invasive carcinoma. (a) Screening mammogram in case 1. Grouped calcification in the middle 3rd of the upper-outer left breast. (b) Magnified image of calcifications from screening mammogram of case 1. (c) Diagnostic mammogram confirming the findings in screening mammogram (case 1). (d) Screening mammogram in case 2. There appear to be grouped developing calcifications in the middle 3rd of the upper-outer left breast. (e) Magnified image of calcifications from screening mammogram of case 2. (f) Diagnostic mammogram confirming the findings in screening mammogram (described as grouped coarse heterogeneous calcifications (case 3)). (g) Diagnostic mammogram performed for high-risk screening. Grouped calcifications are seen in the retroareolar right breast, in the anterior to middle 3rd depth (case 3).

Figure 2 demonstrates representative images of a patient with upstaging from LCIS to invasive carcinoma. The imaging characteristics of all three upgraded patients were predominantly defined by calcifications rather than a mass. The calcifications were defined as “coarse heterogenous” calcifications, which are by definition a Breast Imaging Reporting and Data System (BI-RADS) 4B lesion [11]. BI-RADS 4B lesions on mammography are lesions that have a 10–50% risk of malignancy. LN, in these situations, is concordant with coarse heterogeneous calcifications. There is no one radiological finding that can differentiate LN from more invasive carcinomas or DCIS.

## 4. Discussion

Approximately 39 million mammograms are performed in the US yearly [12]. As the quality of mammographic screening has improved and additional screening such as ultrasound and MRIs are also increasing in use, it is inevitable that biopsy rates will also increase. It has previously been estimated that after routine screening, approximately 10% of patients will need a biopsy. Of those biopsied, 49.4% had second procedures, 20.1% followed with third procedures, and 10.0% had a fourth procedure [13].

With large numbers of benign biopsies being done and many of these showing risk markers such as ADH and LCIS, it is important to periodically reevaluate appropriate management of these lesions. As of 2017, as per the American Joint Commission on Cancer, classic LCIS on CNB is deemed a benign finding, with no further recommendations for diagnostic or therapeutic intervention [14]. There is still variability in how this finding is managed with some recommending surgical excision and others recommending radiological surveillance as the best practice for managing LCIS patients [9].

For patients with non-classic LCIS (pleomorphic and florid) on core biopsy, the rate of upgraded diagnosis to malignancy has been reported to be as high as 36% [15]. Patients with pleomorphic LCIS, in particular, are treated by some surgical oncologists with the same approach as for DCIS with complete excision, negative surgical margins, and postoperative radiation. Although this approach is not specifically supported by NCCN guidelines, it is endorsed by the European Society of Medical Oncology (ESMO) guidelines [2, 16, 17]. However, as outcome data regarding treatment for pleomorphic and florid LCIS are lacking, a multidisciplinary case-based approach should be employed to agree on a treatment course for each patient [16].

A meta-analysis of 9 studies showed limited generalizability and significant uncertainty among LCIS management guidelines [10]. Diagnosis upstaging from LCIS to invasive breast cancer or to ductal carcinoma in situ ranges from 2% to 25% [10]. Metovic et al. reported 28.3% in ALH, LCIS, and high-grade LN cases [18], whereas Singh et al. reported 4% upgrade in classic LCIS, which is similar to our own observations [19]. Laws et al. have reported a series of 77 patients with 78 LN lesions, primarily treated with conservative management rather than surgical excision [20]. They demonstrated a 6.2% risk of conservative management failure. Thus, when conservative management is chosen, careful follow-up with imaging is needed.

Although the percent of upstaging varies between reviews, there are themes regarding certain characteristics that are atypical for LCIS that, when found, warrant surgical excision as they are associated with an increased risk of upstaging [21]. A mass lesion, whether found radiographically or on physical exam, is generally inconsistent with LCIS and its presence makes further diagnostic workup more justifiable [21]. Conversely, specific factors such as a lesion size less than 1 cm in combination with the absence of residual calcifications after biopsy were consistent with benign disease and the absence of upstaging [22]. In addition, certain patient characteristics that may predispose them to malignancy, such as family history, may be considered when deciding whether or not to pursue surgical intervention [23]. Recent guidelines from the American Society of Breast Surgeons recommend observation for LCIS and ALH diagnosed on CNB only if a specific set of criteria is met: that there is concordance between the imaging and pathology results, the lesions are small volume without atypia or other high-risk features, and that serial follow-up and repeat imaging are performed [24]. There is increasing evidence that careful surveillance is a reasonable alternative to excisional biopsy in the majority of patients with LN on core biopsy [25]. However, in this retrospective study, we found that 5% of our cohort who met the abovementioned criteria of close follow-up had an upstage of their diagnosis. The majority (91%) of these diagnoses were made on routine mammographic screening, which can impact the follow-up required thereafter [26]. An additional 17% of cases showed a change in diagnosis to pleomorphic LCIS or ADH, which can change the further management of these patients. We recommend that, in such cases, further risk factors should be considered, surgical excision should be offered to these patients, and shared decision making with the patient should be an integral part of management. Family history enters into the decision-making process, and patients with a family history of breast cancer may be more anxious to proceed with biopsy as opposed to observation. As noted above, 26% of our patients had a first degree relative (mother or sister) with breast cancer and 60% had a second or third degree relative with breast cancer.

We acknowledge that our study has some limitations. We do not have additional information on the 2 patients who were lost to follow-up. Even though there was no evidence of missing the radiological targets, we cannot completely exclude the chances of a false negative, and thus close surveillance of these patients is warranted [27]. In addition, any patients with radiological-pathological discordance were excluded, as that would warrant further surgical workup. Due to the study's retrospective nature, we were also unable to review all of the histopathological slides of these patients. However, the reports were verified before analyzing the results.

## 5. Conclusion

Our data show that 5% of patients, initially shown to have LN by CNB, and who then underwent excisional biopsy, were found to have malignant tumors. Careful radiologic and pathologic review of each case as well as consideration of family history and other risk factors is needed to evaluate whether to recommend excision or close surveillance. Decision to undergo surgical excision should be made through shared decision making with the patient, with the benefits of early detection weighed against the risks of undergoing an invasive procedure.

## Figures and Tables

**Table 1:** Patient characteristics and outcomes.

Variable, *n* (%) unless otherwise stated	ALH (*n* = 61)	LCIS (*n* = 5)	ALH + LCIS (*n* = 2)	Total (*n* = 68)
Age				
Mean	55	51	55	55
<50 years	18	3	0	21
≥50 years	43	2	2	47
BMI, mean	26.0	27.5	25.4	25.7
Race				
White	48	4	1	53
Black	3	0	0	3
Asian	3	1	0	4
Other/unknown	7	0	1	8
Ethnicity				
Hispanic	6	0	0	6
Non-Hispanic	52	5	2	59
Other/unknown	3	0	0	3
Imaging				
Mammogram	54	4	2	60
MRI	6	1	0	7
Ultrasound	1	0	0	1
Follow-up:				
Excisional biopsy	51	5	2	58
Surveillance	8	0	0	8
Lost to follow-up	2	0	0	2
Excisional biopsy results				
Benign	2	0	0	2
Atypia (unspecified)	1	0	0	1
ALH/classic LCIS	35	4	1	40
ADH	8	0	1	9
Pleomorphic LCIS	1	0	0	1
DCIS	2	0	0	2
Invasive carcinoma	0	1	0	1
Change in diagnosis	13	1	1	15/58 (25.9%)
Cancer diagnosis	2	1	0	3/58 (5.2%)

ALH: atypical lobular hyperplasia; ADH: atypical ductal hyperplasia; LCIS: lobular carcinoma in situ; DCIS: ductal carcinoma in situ.

**Table 2:** Radiology and pathology features of patients with cancer diagnosis or pleomorphic LCIS on excision.

Presentation	Details of pathology and radiology findings
75-year-old non-Hispanic white female Routine screening mammogram Family history: maternal aunt with breast and ovarian cancer	Screening mammogram: new grouped calcifications, confirmed on magnification views Core biopsy: ALH Excisional biopsy: DCIS, solid and cribriform types, intermediate nuclear grade (grade 2) with necrosis (focal), and no calcification is present in 4/14 blocks Extent (size) of DCIS: Number of blocks with DCIS: 4 Number of blocks examined: 14 Margins were uninvolved by DCIS Distance from the closest margin in millimeters (mm): <1 mm from the inferior and posterior margins, 2 mm from the medial margin, and >2 mm from the remaining margins (mm) Calcifications present in non-neoplastic tissue AJCC classification (8^th^ edition): pTis, pNx ER+

53-year-old non-Hispanic white female Routine screening mammogram Family history: mother with bilateral breast cancer in her 50s	Screening mammogram: new grouped calcifications, confirmed on magnification views Core needle biopsy: LCIS Excisional biopsy: multifocal invasive carcinoma with ductal and lobular features, grade 2 Tumor size: greatest dimension of largest focus of invasion >1 mm: 3 mm Histologic grade (Nottingham histologic score) *Glandular (acinar)/tubular differentiation* Score 3 (<10% of tumor area forming glandular/tubular structures) *Nuclear pleomorphism*: Score 3 (vesicular nuclei, often with prominent nucleoli, exhibiting marked variation in size and shape, occasionally with very large and bizarre formed) *Mitotic rate*: Score 1 (*N*/ = 3 mitoses per mm^2^) *Overall grade*: Grade 2 (scores of 6 or 7) Tumor focality: multiple foci of invasive carcinoma No DCIS present. LCIS present Staging: IA: mpT1a, N0 ER+ PR+ HER2−

53-year-old Asian female Six-month follow-up evaluation of right breast calcifications Family history: maternal aunt with breast cancer	Mammogram: grouped faint calcifications Core biopsy: ALH Excisional biopsy: DCIS, solid, cribriform, and clinging types (intermediate to high nuclear grade; grade III) with calcifications and single-cell necrosis present in the area of and away from the biopsy site changes in 9 of 15 blocks, spanning approximately 1.8 cm. The biopsy also showed LCIS with calcifications, ADH, and scattered numerous benign calcifications Size (extent) of DCIS: greatest dimension: 18 mm Number of blocks with DCIS: 9 Number of blocks examined: 15 Distance from the closest margins: DCIS was <1.0 cm from the anterior, lateral and superior margins and >0.2 cm from the remaining margins Calcifications: present in DCIS, non-neoplastic tissue, and LCIS AJCC classification (8^th^ edition): pTis, pNX ER+

72-year-old non-Hispanic white female Routine mammogram screening showed left breast calcifications for which she underwent six-month follow-up evaluation Family history: sister was diagnosed with breast cancer at age 38 Personal history: she has a history of 3 benign surgical biopsies and benign needle biopsies which were consistent with atypia	Mammogram: grouped calcifications Core biopsy: atypical lobular hyperplasia with apocrine features (non-classical) involving adenosis and associated with few small calcifications Excisional biopsy: lobular neoplasia (lobular carcinoma in situ), pleomorphic type involving sclerosing adenosis. Biopsy site changes. The pleomorphic type lobular neoplasia (lobular carcinoma in situ) is present at the red (superior), at the green (anterior), and <0.1 cm to the black (posterior) margin

CNB: core needle biopsy; ALH: atypical lobular hyperplasia; ADH: atypical ductal hyperplasia; LCIS: lobular carcinoma in situ; DCIS: ductal carcinoma in situ.

**Table 3:** Clinical and pathological features of patients with change in diagnosis to ADH on excision.

Sr no	CNB	Association of calcification with ALH	Excisional biopsy results	Final pathology	Association of calcifications	Family history
1	ALH	None	ADH + LCIS	LCIS; FEA with calcium; ADH; biopsy site changes	FEA	Two paternal aunts: breast cancer in their 60s
2	ALH	None	ADH	Cellular fibroadenoma with ADH. ALH, cystic changes, biopsy site changes	None	None
4	ALH	None	ADH	ADH; ALH; FEA with focal calcifications; biopsy site changes; usual ductal hyperplasia; radial scar; pseudoangiomatous stromal hyperplasia; columnar cell change with focal calcifications; apocrine metaplasia and cysts	FEA; columnar changes	Sister: uterine cancer
5	ALH	None	ADH	ALH; focal ADH; biopsy site changes completely excised; secretory changes, apocrine metaplasia, and columnar cell change; rare benign calcifications	Benign	Brother: CDH1 pos; mother: breast cancer; father: gastric cancer; cousins and paternal grandfather: gastric cancer
6	ALH	None	ADH	Focal ADH; ALH; fibroadenomatous nodule; biopsy site changes, completely excised; scattered benign calcifications in association with normal lobules and cysts; additional findings include columnar cell change, sclerosing adenosis, and cysts	Benign	Maternal grandmother: breast cancer
7	ALH + LCIS	Yes	ADH + LCIS	Severe ADH, focal; LCIS and ALH, multiple foci; sclerosing adenosis, focal, with microcalcifications; PASH; cysts; apocrine metaplasia; focal benign microcalcifications; biopsy site changes	Benign	Mother: breast cancer and bile duct cancer; maternal grandmother: colon cancer
9	ALH	None	ADH	Severe ADH; FEA; ALH; microscopic radial scar with calcifications; columnar cell change; biopsy site changes	Microscopic radial scar	None
10	ALH	None	ADH	ADH; apocrine metaplasia and hyperplasia with apocrine atypia; ALH; usual ductal hyperplasia, cysts; scattered small calcifications mostly with non-atypical areas, including the pinned area; changes consistent with previous biopsy site	Non-atypical areas	Brother: stomach cancer
11	ALH	None	ADH	ADH; ALH; intraductal papillomas and marked duct ectasia; biopsy site changes	None	Mother: IDC, triple +; maternal great-grandmother: breast cancer; paternal grandfather: lung or colon cancer

CNB: core needle biopsy; ALH: atypical lobular hyperplasia; ADH: atypical ductal hyperplasia; FEA: flat epithelial atypia; IDC: invasive ductal carcinoma; LCIS: lobular carcinoma in situ.

## Data Availability

Deidentified data can be requested from Dr. Pories at spories@mah.harvard.edu. Release of data would need to be approved by our IRB.
